# Function, Evolution, and Composition of the RpoS Regulon in *Escherichia coli*

**DOI:** 10.3389/fmicb.2020.560099

**Published:** 2020-09-17

**Authors:** Herb E. Schellhorn

**Affiliations:** Department of Biology, McMaster University, Hamilton, ON, Canada

**Keywords:** regulation, sigma factor, adaptation, pathogenesis, evolution

## Abstract

For many bacteria, successful growth and survival depends on efficient adaptation to rapidly changing conditions. In *Escherichia coli*, the RpoS alternative sigma factor plays a central role in the adaptation to many suboptimal growth conditions by controlling the expression of many genes that protect the cell from stress and help the cell scavenge nutrients. Neither RpoS or the genes it controls are essential for growth and, as a result, the composition of the regulon and the nature of RpoS control in *E. coli* strains can be variable. RpoS controls many genetic systems, including those affecting pathogenesis, phenotypic traits including metabolic pathways and biofilm formation, and the expression of genes needed to survive nutrient deprivation. In this review, I review the origin of RpoS and assess recent transcriptomic and proteomic studies to identify features of the RpoS regulon in specific clades of *E. coli* to identify core functions of the regulon and to identify more specialized potential roles for the regulon in *E. coli* subgroups.

## Introduction

*Escherichia coli*, like many free-living bacteria, lives a biphasic lifestyle that consists of alternating periods of rapid growth and nutrient deprivation. These periods may be accompanied by stresses such as desiccation and other adverse chemical/physical conditions including osmotic stress, nutrient deprivation, oxidative stress and acid stress. These diverse environmental challenges require coordinated sensing and response through programmed changes that include efficient physiological adaptation and reprogrammable modulation of gene expression. This can be accomplished, in populations, by evolutionary selection for favorable traits that enhance survival and, in individual cells, through the activation of specific regulatory processes that allow the cell to adapt to new metabolic and physical challenges. However, the initiation of major regulatory systems needed for adaptation often requires a substantial metabolic commitment required for the *de novo* expression of a large number of new proteins. Therefore, global control of gene expression must be finely tuned to the specific needs of the cell. For example, stationary phase adapted cells alter macromolecular biosynthesis and nutrient utilization strategies to survive potentially hostile environments. Much of our knowledge of bacterial regulation has come from countless studies of a few laboratory *E. coli* strains grown under laboratory conditions that, while useful, probably imperfectly mimic bacterial growth in the natural environment.

This review examines features of the RpoS regulon from a functional and evolutionary perspective and will thus not include a consideration of the many factors that modulate the regulation of RpoS itself. Recent reviews cover other specific aspects These include small RNAs (Fröhlich and Gottesman, 2018), proteolysis, relationship to other sigma factors, other stress response systems (Gottesman, 2019) and relationship to the global transcriptional machinery (Ishihama, 2018). The important mechanistic interaction of the small Crl protein with RpoS is covered in detail elsewhere (Cavaliere and Norel, 2016). Earlier reviews have examined the functional aspects of the RpoS regulon (Landini et al., 2014; Schellhorn, 2014).

## RpoS Evolution

Bacterial regulons are generally plastic (Lozada-Chavez et al., 2006) adapting to niche-specific needs of the bacterium. As an adaptive regulator, RpoS is not essential for the core metabolic functions of the cell. However, given its many potential ancillary roles in stress responses, the evolutionary emergence of RpoS as an alternative, non-essential vegetative sigma factor, undoubtedly provided new regulatory possibilities to descendant groups of bacteria. Sequence similarity and gene synteny indicate that RpoS likely arose through an RpoD duplication event prior to the emergence of the Proteobacteria (Chiang and Schellhorn, 2010) followed by loss of the large N-terminal 1.1 region of RpoD in one of the two RpoD paralogs to yield a truncated RpoS protein. It is thus found only in the Proteobacteria (Chiang and Schellhorn, 2010). Gene synteny and sequence differences indicate that this Proteobacterial RpoS is distinct from the *Borrelia* RpoS, which arose independently of the proteobacterial RpoS-RpoD duplication event. Why RpoS developed is an interesting evolutionary question and this may because many members of the gamma proteobacteria have distinct biphasic lifestyles in which they live as either free organisms or in association with a host.

Comparisons of the RpoS regulons of *E. coli* and *Pseudomonas* spp. reveal that, while there are conserved core functions within the regulon, these represent less than 25% of the RpoS orthologs shared between these organisms (Chiang and Schellhorn, 2010) As several orthologs are thought to have diverged before the RpoS-RpoD duplication event, it is likely that genes were/are recruited into the regulon through selective pressure (Chiang and Schellhorn, 2010). Other regulons “recruitments” have occurred more recently and have likely included horizontal gene transferred (HGT) functions (Dong and Schellhorn, 2009). Consistent with this idea, many genes in O pathogenicity islands are RpoS-dependent (Dong and Schellhorn, 2009).

RpoS works in concert with the small Crl protein to modulate RpoS regulon expression (Typas et al., 2007). Crl can function as a either negative or positive cofactor to modulate expression of distinct subsets of the RpoS regulon and is a particularly potent effector when RpoS levels in cells are low (Typas et al., 2007). This likely explains why some RpoS regulon members are expressed in exponential phase (Dong et al., 2008) when levels of RpoS are extremely low (Dong and Schellhorn, 2009). Interestingly, the Crl protein is conserved within and restricted to the GammaProteobacteria (Cavaliere et al., 2015; Santos-Zavaleta et al., 2019) but as two variants: one that directly contacts RpoS to facilitate formation of the RpoS-RNA polymerase complex formation and a second that does not make direct contact with RpoS and therefore does not play a role in RpoS modulation of gene expression) (Cavaliere et al., 2015). As it is less widely distributed than RpoS, which has a broader distribution in the Proteobacteria (above), it probably evolved as an accessory regulator after RpoS/RpoD divergence (Santos-Zavaleta et al., 2019).

The RpoS regulatory system thus represents an adaptable system that plays slightly different physiological roles subgroups (classes) of the proteobacteria depending on physiological needs and these can include adaptation to hosts to adaptation to nutrient-deprived environments where cells may encounter physical and chemical stresses that are not part of the typical host environment.

While many bacterial gene regulation studies have employed exponential phase cultures, examining bacterial adaptation to stationary phase in laboratory culture may be a useful proxy for understanding how bacteria transition to suboptimal growth conditions in the natural environment. During stationary phase adaptation, the *E. coli* cell undergoes morphological remodeling (Lange and Hengge-Aronis, 1991), becomes resistant to specific stresses (e.g., heat and oxidative stress; Vidovic et al., 2012; Mata et al., 2017), and substantially reduces overall macromolecule biosynthesis (Yoshida et al., 2018). Translation is down-regulated by the dimerization of ribosomes from the active 70S form to a quiescent 100S form (Yoshida et al., 2019). Transcription, though also reduced in stationary phase, is altered by the effective displacement of the major housekeeping RpoD sigma factor by the minor RpoS sigma factor, which, in coordination with many other protein and RNA factors, initiates the expression of a large complex regulon. This is followed by structural changes in the *E. coli* cell, including condensation of the nucleoid (Azam et al., 2000), morphological transition to rounded cells (Lange and Hengge-Aronis, 1991) and an increase in compatible solute synthesis (Hengge-Aronis et al., 1991). The RpoS regulon in *E. coli* includes hundreds of genes that require a large metabolic commitment in terms of RNA and protein synthesis. Therefore, it must have evolutionarily adapted to the specific metabolic requirements of *E. coli* cells to confer a selective advantage. In contrast, other proteobacterial lineages (e.g., alpha and epsilon proteobacteria) have lost RpoS function altogether during their evolutionary history (Chiang and Schellhorn, 2010).

## RpoS Regulon of *Escherichia coli* K12

In *E. coli* and related bacteria, the RpoS regulatory system has become a paradigm for global adaptation since its discovery (Mulvey and Loewen, 1989). Initially identified as a regulatory sigma factor controlling a few stress genes, RpoS is now recognized as an important multifaceted control system in many proteobacteria regulating many diverse processes including nutrient scavenging, expression of virulence factors, acid resistance, osmotic stress resistance, and synthesis of cell structural components. A large fraction of the bacterial genome is positively controlled by RpoS (Lacour and Landini, 2004; Patten et al., 2004; Weber et al., 2005) and many genes are negatively controlled (Patten et al., 2004). Despite its general role in adaptation, loss of RpoS function mutations may be beneficial in some cases and may lead to enhanced nutrient utilization. This potential benefit may explain how a selective pressure for loss of RpoS may have occurred in some proteobacterial lineages (Chiang and Schellhorn, 2010) and to the accumulation of RpoS loss of function in individual *E. coli* laboratory strains. RpoS can be highly polymorphic (variable in expression or activity) in environmental isolates and loss of RpoS can be experimentally selected in pathogenic *E. coli* (Dong et al., 2009a). Laboratory domestication of natural isolates may lead to the acquisition of *rpoS* attenuation mutations (Bleibtreu et al., 2014), underscoring the need for careful handling during cultivation (including minimizing freeze thaw cycles and frequently checking RpoS phenotype).

RpoS levels are low in exponential phase and increase several-fold as cells enter stationary phase (Lange and Hengge- Aronis, 1994). This increase is regulated by many factors including small RNAs, ClpX-mediated proteolysis, and interactions with other proteins (see Gottesman, 2019, for review). Thus RpoS function in exponential phase is reduced both by low concentrations of the protein (Tanaka et al., 1993) and by interactions with anti-sigma factors (Jishage and Ishihama, 1999; Yoshida et al., 2019). Nonetheless, interaction through Crl-mediated control allows several exponential phase genes to be expressed (Dong et al., 2008). The large size of the RpoS regulon made it an early candidate for study using transcriptomic technology with estimates of the number of RpoS-controlled functions of 400–500 genes (Lacour and Landini, 2004; Patten et al., 2004; Weber et al., 2005). As many genes are organized in operons or indirectly controlled through the action of RpoS-controlled regulators, the number of promoters actually directly recognized by RpoS is much lower. Transcriptomic technology (RNA-Seq and/or microarray), in itself, an only reveal whether genes are controlled by a given regulator. It does not indicate, however, whether the observed regulation is direct (regulator acting directly on target promoters) or indirect (regulator acting on the promoter of an intermediate regulator).

Overexpression of genes controlling key metabolic pathways, particularly the TCA cycle (Patten et al., 2004), may be important for nutrient scavenging in RpoS-attenuated cells and may reduce gene expression. There are at least two means by which RpoS may have a negative regulatory role: (1) through sigma factor competition for core polymerase (Farewell et al., 1998), and (2) through RpoS/RpoD competition for stationary phase promoters (Cho et al., 2014). The latter can be explained by the fact that some promoters are also recognized by RpoD which can have a higher affinity for RpoS promoters than RpoS itself leading to “up-regulation” of RpoS dependent promoters in stationary phase (Cho et al., 2014). Thus, In the absence of RpoS, RpoD, which is present in high amounts in stationary phase, may functionally substitute for RpoS to express several stationary RpoS-dependent phase genes (Cho et al., 2014).

**FIGURE 1:**
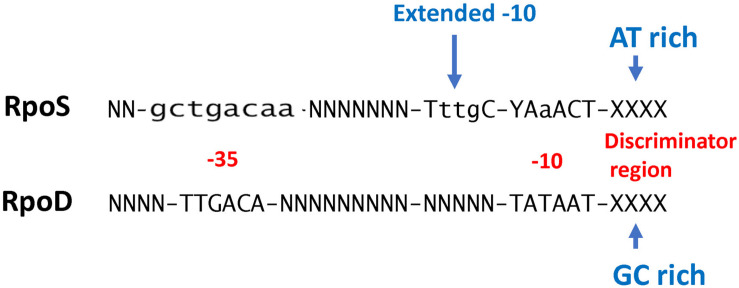
Comparison of RpoS and RpoD consensus promoter recognition sequences (W = A or T; R = A or G; N = any base). RpoD sequence information is from Shimada et al. (2017) and RpoS sequence information is from Peano et al. (2015).

While conventional transcriptome studies using microarrays or RNA-SEQ provide a global overview of gene regulation, the use of ChIP-SEQ combined with RNA-SEQ and DNA sequence localization technologies can more precisely determine the numbers and identities of promoters and their binding affinities for RpoS to identify sequence determinants and better understand the relationship between RpoS and the regulon that it controls. Several groups (Cho et al., 2014; Peano et al., 2015; Wong et al., 2017; Table 1) have employed this approach and several generalizations regarding the nature of the regulon can be made. These studies extend the idea that (1) RpoS directly controls over 1000 genes in *E. coli* with about 2/3 being positively controlled and the remainder being negatively controlled (Cho et al., 2014; Wong et al., 2017); and (2) DNA binding sites for RpoS are consistent with the previous promoter consensus sequence predictions, namely that there is a consensus −10 promoter sequence with a C at the −13 position in an “extended” −10 sequence, an AT-rich discriminator region and a weak −35 consensus sequence (Peano et al., 2015; Wong et al., 2017; Figure 1). The total number of more than 1000 targets includes both direct and indirect targets. The total of 129–179 core promoters of RpoS were identified *in vitro* using the qSELEX screening system (Shimada et al., 2017).

**TABLE 1:** Size and nature of the RpoS regulon in *Escherichia coli* based on ChIP-SEQ, RNA-SEQ, Mobility Shift assays, and qPCR.

		**DNA Binding sites^*a*^**			**Promoters^*b*^**			**Genes^*c*^**			
**Group**	**Methodology**	**Total**	**Intergenic**	**Intragenic**	**Targets**	**Positive**	**Negative**	**Targets**	**Positive**	**Negative**	**Notes**
Cho et al. (2014)	ChIP-SEQ/Microarray	1139^*d*^			1139	903	178	1139^*e*^	291	178	Used multi-sigma factor binding to identify non-canonical binding, microarrays to correlate positive and negative control
Peano et al. (2015)	ChIP-SEQ/RT-PCR/GMSA	78	61	2	63	50					Found binding does not correlate with binding affinity
Shimada et al. (2017)	Genomic Selex enrichment	218	125	73	129–179						Promoter assignment based on gene proximity and orientation
Wong et al. (2017)	ChIP-SEQ/RNA-SEQ/qPCR	286	217	67				1044	605	439	Identified three classes of promoters based on sensitivity to RpoS levels

*^a^Determined by ChIP-SEQ or by Selex enrichment of RNAP-bound sequence.^b^Identified by proximity to and orientation of potential open reading frames. ^c^Positive/negative control determined by WT vs. RpoS null expression comparisons.^d^Of these, 903 are uniquely bound by RpoS, the remainder were also bound by other sigma factors.^e^While 1139 genes were assayed, 670 genes were not significantly different in expression.*

Strain variability has been a longstanding problem that continues to make it difficult to make generalizations regarding RpoS control or the composition of the RpoS regulon especially since most studies have only examined expression in small number of laboratory strain backgrounds. Even strains derived from a single parental stock can exhibit substantial variability in levels of RpoS (Jishage and Ishihama, 1997; Liu et al., 2017). Identifying bona fide regulon members using transcriptomic technologies is complicated by sigma factor competition for core polymerase, variable binding affinities of sigma factors for cognate promoters, variable concentration of sigma factors themselves and the modulation of sigma factor recognition and binding by accessory factors. Comprehensive estimates of sigma factor concentration under physiological conditions (Jishage and Ishihama, 1995) and determination of binding constants (Maeda et al., 2000) will aid in the development of robust models of regulon expression (Ishihama, 2018).

RpoS promoters can be classified, using araBAD-controlled RpoS expression system (Wong et al., 2017), as either sensitive or relatively insensitive to activation during adaptation to stationary phase. Sensitive promoters are highly responsive to slight increases in intracellular RpoS levels while insensitive promoters exhibit a lagging response (Wong et al., 2017).

Negative control of RpoS-controlled genes may be direct through physical contact between Crl and RpoS (Levi-Meyrueis et al., 2015). Transcriptome data comparisons of wildtype vs. *rpoS* null mutants indicate a large number of affected genes, but many of these are only weakly negatively regulated and are probably only scored as such because transcriptome data is normalized to an invariant gene set. If a large amount of RNA is scored as positively regulated in the wildtype type, an equal amount of RNA is scored as overexpressed in the mutant. Nonetheless, entire genetic pathways/systems are negatively controlled in *E. coli* including the TCA cycle, flagellar biosynthesis, and cryptic prophage genes (Patten et al., 2004). The TCA cycle and motility (flagella) can be understood in the context of stationary phase physiology as they may allow RpoS null mutants cells to more efficiently utilize nutrients such as amino acids and organic acids that feed into the TCA cycle. The benefit of overexpression of cryptic phage genes is not immediately obvious, but these genes appear to enhance metabolic capabilities of the cell in late stationary phase. For example, *E. coli* possesses nine cryptic prophage clusters whose expression increases in stationary phase, and this expression is enhanced in *rpoS* mutants (Wang et al., 2010). Combinatorial deletion of these prophage elements reduces the range of nutrients that the cell can use and renders the cell sensitive to stress (Wang et al., 2010).

While most growth phase studies have focused on early stationary phase adaptation (>2 days) of *E. coli*, *E. coli* is viable for much longer periods in a presumably senescent state. Intriguingly, one of the few proteomic studies on extended cultures indicates that unique protein profiles, not expressed in either exponential or early stationary phases, are expressed up to at least 8 days of culture (Yoshida et al., 2019). The role of RpoS (or other regulators) in the expression of these proteins, several of which have predicted repair functions (Yoshida et al., 2018) has not yet been examined.

## RpoS as a Metabolic Switch

Two hypotheses regarding the possibility that RpoS may be a central regulator in a stress-vs.-nutrition paradigm were suggested by the Zinser and Kolter (2004) and Ferenci labs (Ferenci and Spira, 2007). The first proposed that mutant subpopulations developed in stationary phase that possessed a competitive Growth Advantage in Stationary Phase (GASP) relative to wildtype cells based on enhanced nutrient-scavenging capabilities (Zinser and Kolter, 2004). Consistent with this idea that *rpoS* mutations can be beneficial, it has been suggested (Robinson et al., 2020) that *rpoS* mutants have a selective advantage in mixed culture by functioning as “cheaters” in that they benefit for the products produced by the wildtype but do not have to pay the high metabolic cost of expressing the large RpoS regulon. A second, slightly different, hypothesis suggests that bacteria that, like *E. coli*, faced with stress factors or enhanced nutritional sources allocated resources to ensure maximum viability through Self-Preservation and Nutritional Competence (SPANC) (King et al., 2004; Ferenci and Spira, 2007). Functional RpoS allows the cell to allocate resources to counter stress or, through selection for loss of RpoS function mutations, enable the cell to utilize an expanded range of substrates. This may explain how, in many situations, RpoS loss of function mutations can contribute to improved nutrient scavenging or, in some cases, through host adaption, to enhanced pathogenesis (Zlatkov and Uhlin, 2019).

Transcriptome and proteome studies assess global expression in a population rather than activities within a single cell. However, RpoS levels are heterogeneous among single cells in a population (Patange et al., 2018), suggesting that stochastic variation may be an important determinant in generating subpopulations of cells within a population. This may result in a given population having a far greater range of capacities to survive stress and have extended nutrient utilization capacity than a homogenous population. A role for RpoS would be consistent with the observation that bacteria form subpopulations in stationary phase that are distinct from those in exponential phase (Yoshida et al., 2018). The use of fluorescent reporter expression systems should help us to better understand how altered gene expression in single cells allows bacteria to adapt to changing environments (Patange et al., 2018).

## Variability of RpoS in Other Strains of *E. coli*

*E. coli* is a highly adaptable opportunistic pathogen that can colonize hosts through the horizontal acquisition of virulence factors and modulation of the function of global regulators such as RpoS. In enterohemorrhagic *E. coli* O157:H7, core RpoS-stress adaptation functions as well as key metabolic pathways, important for intestinal colonization, are controlled by RpoS (Dong and Schellhorn, 2009). The latter include arginine degradation, fatty acid oxidation, and polyamine cycling. Relative to *E. coli* K12, O157:H7 has a much larger genome (5.5 vs. 4.6 Mb) with much of the additional DNA located in O-islands many of which encode virulence factors. One such factor is the Locus of Enterocyte Effacement (LEE) operon that encodes bacterial functions needed to produce the Attaching and Effacing (A/E) attachment lesion during intestinal colonization (Franzin and Sircili, 2015). RpoS positively regulates several LEE-encoded elements including the *ler* regulator, *cesF*, and *eae*, an outer membrane protein needed for virulence (Franzin and Sircili, 2015). In the related pathogen *Citrobacter*, RpoS is required for LEE expression and for full virulence underscoring the importance of RpoS in the colonization process (Dong et al., 2009b). Interestingly, some key pathways that are shared between the K12 and O157:H7 strains are differentially controlled (Dong and Schellhorn, 2009). For example, both chemotaxis protein and flagellar biosynthesis are negatively regulated in K12 but are positively controlled in O157:H7. The TCA cycle is negatively controlled in K12, but in O157:H7 there is little difference in TCA cycle-associated transcript levels between WT and an *rpoS* mutant) (Dong and Schellhorn, 2009) suggesting such control may be strain or clade-specific, as it is laboratory strains. RpoS levels in *E. coli* O157:H7 is strain-dependent (Bhagwat et al., 2006), making it somewhat difficult to make generalizations regarding specific RpoS-dependent pathways. Other types of *E. coli* may also show specific niche adaptation involving RpoS. For example, enteropathogenic *E. coli* (EPEC) (which can cause infant diarrhea), RpoS is, as expected, required for stress resistance but has a differential effect on adherence to epithelial cells that is strain-dependent (Mata et al., 2017).

RpoS regulation of biofilm production is positive in *E. coli* K12 but is negative in O157:H7 strains (Carter et al., 2014). In both EHEC O157:H7 and STEC O111 (Diodati et al., 2016), RpoS contributes to autoaggregation through enhanced fimbriae production in strains attenuated in *rpoS* expression. Though the loss of RpoS can render the cell sensitive to stress, the benefit of increased pathogenesis probably outweighs the cost of loss of fitness. Consistent with this idea, loss of RpoS function can be a pathoadaptive process for uropathogenic *E. coli* (Zlatkov and Uhlin, 2019). Wild-type *E. coli* does not normally use citrate, but in some extraintestinal pathogenic E. coli (ExPEC), acquired *rpoS* mutations allow the cell to reduce RpoS-dependent diGMP levels and increase expression of fimbriae and citrate influx through upregulation of the *citT* transporter (Zlatkov and Uhlin, 2019). This loss-of-function *rpoS* mutation leads to enhanced colonization, citrate utilization and higher citrate-complexed iron transport of which all are important in uropathogenesis. This selection is probably restricted to this group of pathovars as RpoS normally has a positive regulatory role in fimbriae formation through positive c-di-dGMP dependent control of the CsgD regulator, a key regulator of biofilm formation (Weber et al., 2006).

## Future Goals

Though our knowledge of RpoS function has dramatically improved through the use of transcriptomic technologies and other bacterial regulatory systems, many outstanding questions remain. Much of our current understanding of RpoS function is based on studies using laboratory-attenuated strains which, based on studies in other strains and organisms, may not reflect the niche-specific adaptation role that RpoS plays in other organisms. The importance of RpoS mutations in the natural environment is still not satisfactorily resolved. While it is clear that laboratory strains can readily acquire inactivating mutations in *rpoS* in either selective conditions or as an unintended consequence of storage and handling the of role attenuated RpoS in feral strains must be better established. It may be that the rewiring of RpoS regulon expression through attenuation of RpoS activity also has effects on the many regulatory and physiological factors that interact with RpoS. It may be important to examine these in parallel in natural strains to obtain a comprehensive picture of how RpoS functions to regulate adaptation in bacterial systems.

## Author Contributions

HS researched and wrote the manuscript.

## Conflict of Interest

The author declares that the research was conducted in the absence of any commercial or financial relationships that could be construed as a potential conflict of interest.
